# Minor trauma-induced eccrine porocarcinoma of the toe in a patient on long-term carbamazepine: a case report and literature review

**DOI:** 10.3389/fonc.2026.1727762

**Published:** 2026-02-13

**Authors:** Lianbo Yang, Jinghang Lv, Xinghan Zhao, Ziang Zheng, Xinyao Cui, Jiabin Lin, Yifan Wang, Haidong Liang

**Affiliations:** Department of Bone and Soft Tissue Repair and Reconstructive Surgery, The Second Hospital of Dalian Medical University, Dalian, Liaoning, China

**Keywords:** carbamazepine, case report, eccrine porocarcinoma, toe neoplasms, trauma-induced

## Abstract

**Background:**

Eccrine Porocarcinoma is a rare malignant neoplasm of eccrine sweat gland origin. Its diverse clinical presentation often leads to misdiagnosis and delayed treatment. The potential role of systemic factors, such as long-term medication use, in its pathogenesis is poorly understood.

**Case presentation:**

We report a case of a 51-year-old male with a 30-year history of epilepsy on long-term carbamazepine therapy, who developed a refractory ulcerative mass on the left second toe after minor trauma. Toe amputation was performed, and histopathology confirmed Eccrine Porocarcinoma.

**Conclusions:**

This case highlights the importance of considering rare malignancies like Eccrine Porocarcinoma in the differential diagnosis of non-healing acral ulcers. An early biopsy is crucial. The long-term use of carbamazepine in this context is noted, and its potential role as a co-factor in tumor development, possibly interacting with local chronic inflammation, is discussed. Further investigation into the influence of systemic medications on the pathogenesis of such rare tumors is warranted.

## 1 Introduction

Eccrine Porocarcinoma (PC) is a rare malignant adnexal tumor thought to originate from the intraepidermal portion of the eccrine duct (acrosyringium). Since its first description in 1963, reported cases remain limited (1). Its incidence accounts for approximately 0.005% to 0.01% of all cutaneous malignancies, predominantly affecting the elderly (2), with a predilection for the lower limbs and head/neck region (3, 4). At the molecular level, PC is frequently characterized by recurrent gene fusions, most notably involving YAP1, MAML2, or NUTM1, which are believed to drive tumorigenesis and may offer diagnostic utility (5).

Clinically, PC exhibits diverse presentations (6), often manifesting as nodular or ulcerative lesions, PC is frequently misdiagnosed as more common conditions like squamous cell carcinoma, basal cell carcinoma, or chronic infectious granulomas, leading to diagnostic challenges and delayed treatment (7, 8). Notably, PC can arise in areas of old scars or chronic trauma, suggesting that persistent local inflammation and repair processes may play a role in its pathogenesis. Furthermore, the association between altered immune status and PC development remains insufficiently explored.

We report a case of PC occurring on the left second toe of a 51-year-old male, characterized by its direct induction by minor trauma (nail clipping) and the patient’s long-term carbamazepine use. This case aptly illustrates the diagnostic pitfalls of PC and its potential association with trauma and underlying immune factors. Through this report, we aim to heighten clinical awareness of rare acral malignancies and emphasize the decisive role of early biopsy in establishing a definitive diagnosis.

## 2 Case presentation

**Figure 1:**
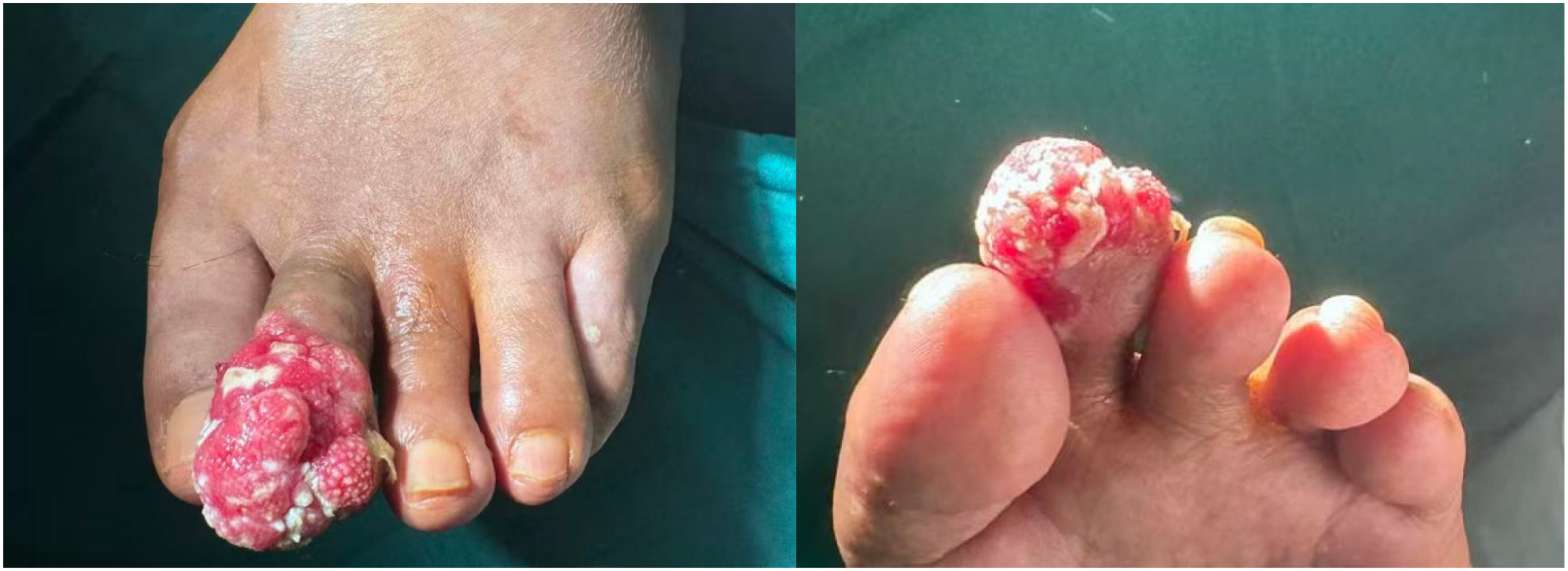
Clinical appearance of the cauliflower-like ulcerated mass on the left second toe.

A 51-year-old male was admitted for a “non-healing ulcer with exudate on the left second toe for over 9 months.” The condition began after accidental skin injury during toenail trimming. The wound failed to heal, gradually enlarged, and was managed by the patient with home dressings without improvement. One month prior to admission, outpatient X-ray revealed soft tissue swelling around the middle and distal phalanges of the left second toe, suggesting possible osteomyelitis (**Figure 1**). Surgery was advised but initially declined by the patient, who opted for continued conservative care with regular outpatient wound care. Due to persistent non-healing, he was admitted for further treatment.

Past medical history was significant for epilepsy of 30 years’ duration, controlled with regular carbamazepine (specific dose: 4 tablets, three times daily), with seizure frequency of 1-2 episodes per month. Other systemic diseases were denied. Nutritional status on admission was good.

**Figure 2:**
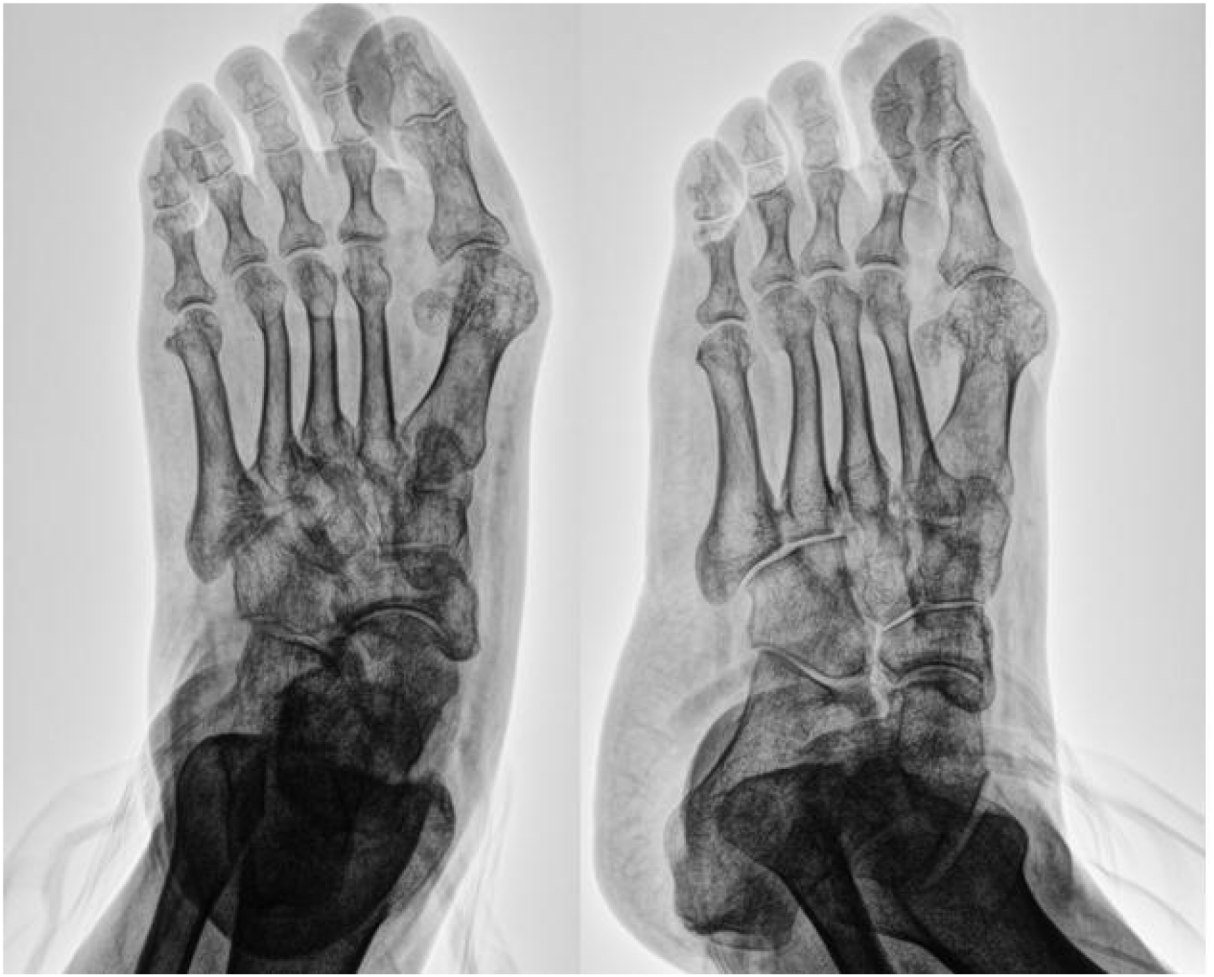
Radiograph of the left second toe showing soft tissue swelling.

Physical Examination: A cauliflower-like ulcerative mass, approximately 3.0 cm in diameter, was observed on the dorsal aspect of the left second toe distal to the proximal interphalangeal joint. The surface was covered with yellowish-green exudate (**Figure 2**).

Laboratory Investigations: Complete blood count showed decreased lymphocyte percentage (15.5%). Biochemical analysis revealed borderline low total vitamin D (20.85 ng/mL) and hypoalbuminemia (34.44 g/L). Coagulation profile, infection markers, liver and kidney function, and electrolytes were within normal limits. The detailed laboratory parameters are summarized in **Supplementary Tables S1–S4**.

Management Course: Given the destructive, chronic nature of the lesion and challenges with complex wound care compliance in an outpatient setting, a left second toe amputation was performed after detailed discussion with the patient and family to achieve disease control and simplify management.

**Figure 3:**
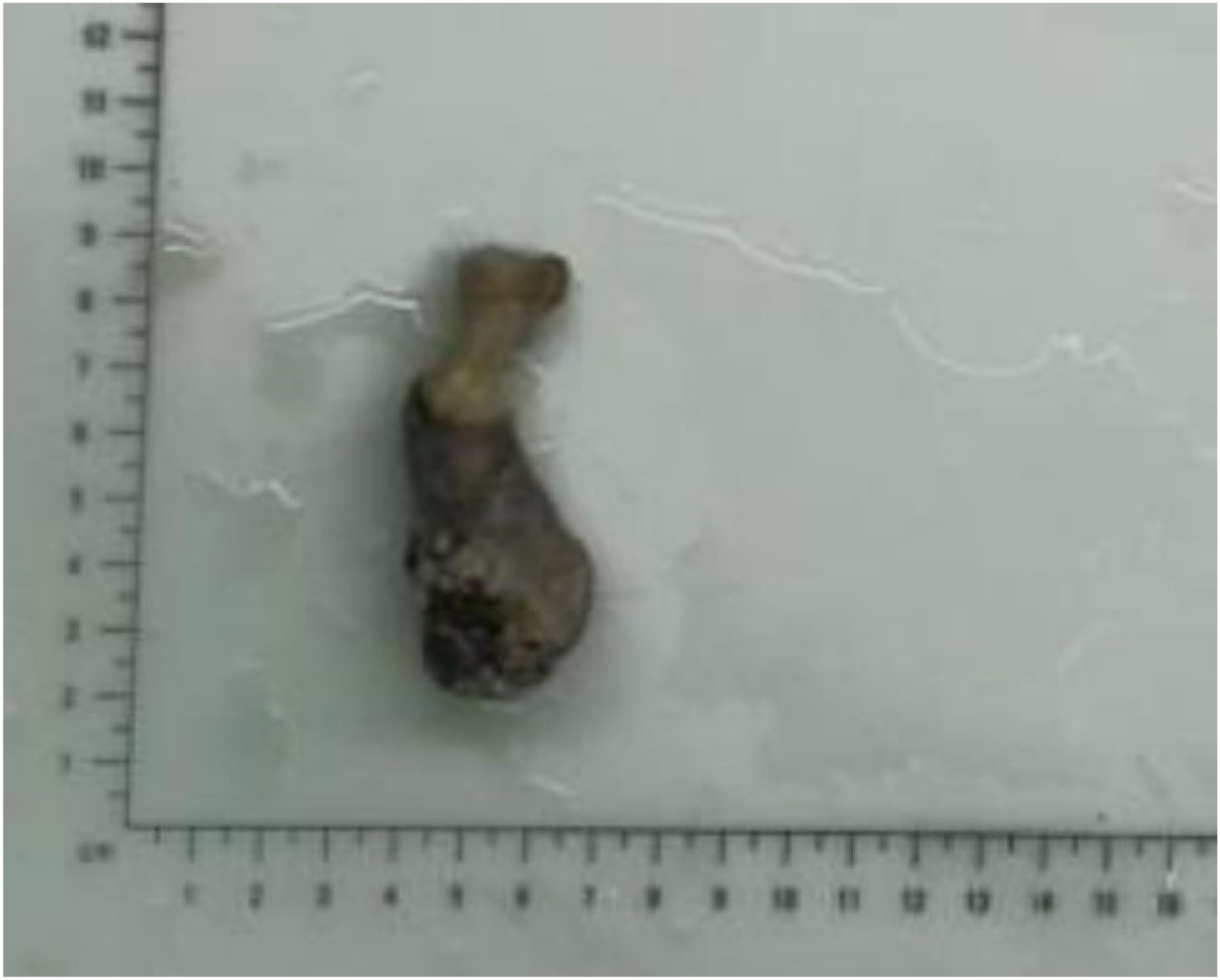
Gross specimen of the amputated toe, illustrating the cross-sectional view of the mass and the adjacent grayish-black area.

**Figure 4:**
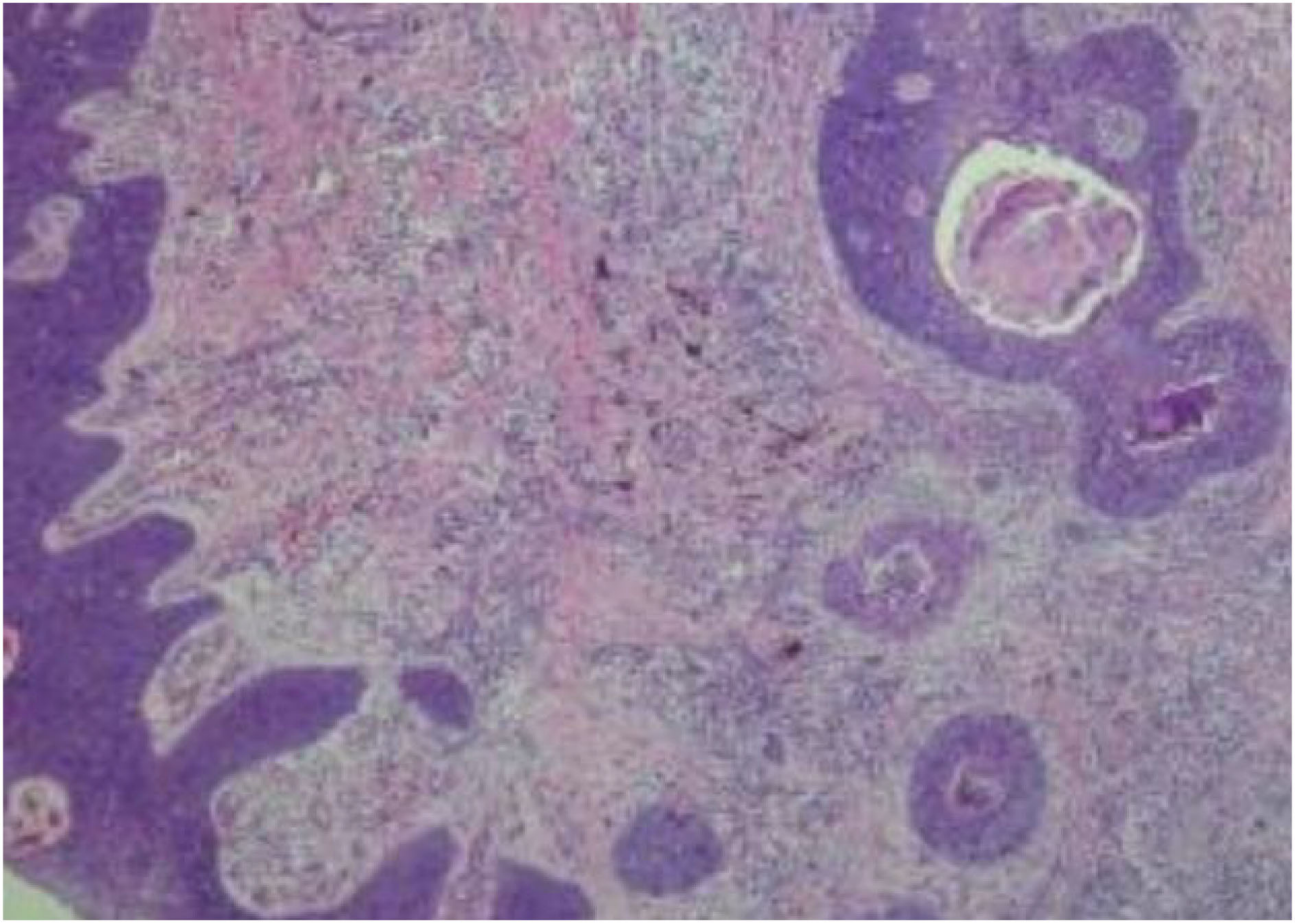
Histopathological examination (H&E staining, original magnification ×40). Multiple, discrete tumor cell nests and cords are observed extending from the upper-right to the lower-left corner of the section. Some of these nests exhibit characteristic lumen-like structures accompanied by necrosis. These morphological features are highly consistent with those of eccrine porocarcinoma, supporting the diagnosis.

Pathological Findings: The gross specimen measured 7.0 × 2.5 × 2.0 cm. A well-demarcated mass (~3.0 × 3.0 × 2.0 cm) was noted distally, with a grayish-red, moderately firm cut surface. An adjacent gray-black area (~2.5 × 2.0 cm) was also present (**Figure 3**). Histopathology revealed a dermal tumor connected to the epidermis. Invasive nests of basaloid cells with significant atypia and frequent mitotic figures were seen. Some nests contained lumen-like structures with focal necrosis (**Figure 4**). The diagnosis was consistent with malignant tumor, morphologically highly suggestive of eccrine porocarcinoma, with an invasion depth of approximately 6 mm. Immunohistochemistry was suggested intraoperatively for definitive diagnosis but was declined by the patient due to financial reasons. All surgical margins were free of tumor involvement.

**Figure 5:**
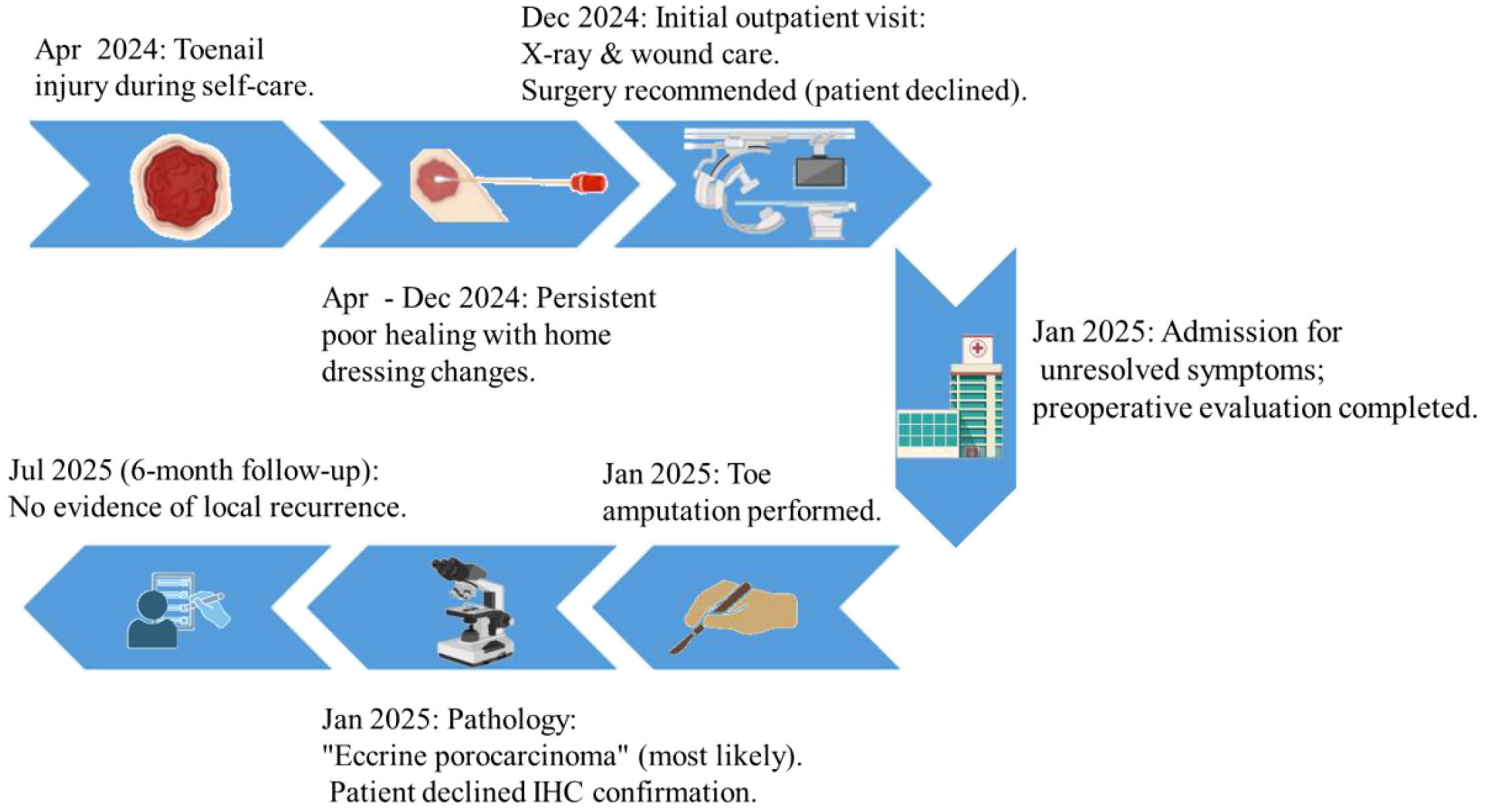
Comprehensive clinical timeline and management pathway. This flowchart illustrates the sequential timeline of events from the initial toe injury, through the phases of conservative management, surgical decision-making and intervention, to the postoperative recovery and 6-month follow-up assessment.

The postoperative course was uneventful. The patient was discharged with scheduled suture removal and referred to a community rehabilitation center for follow-up. At the 6-month follow-up, the surgical site was well-healed with no signs of local recurrence. The entire patient journey, from the initial trauma through treatment decisions to postoperative follow-up, is encapsulated in the clinical timeline (**Figure 5**).

## 3 Patient perspective

The patient’s socioeconomic context and personal values significantly shaped his healthcare decisions. He was unmarried, lived alone, had no children, and faced considerable financial constraints. His long-standing epilepsy, managed with carbamazepine, limited his employment opportunities. The costs associated with this hospitalization were primarily covered by relatives.

This background directly influenced his initial choice. During the first outpatient consultation, despite understanding the proposed surgery’s rationale, his decision was heavily weighted by concerns over additional costs (for the procedure, anesthesia, and postoperative recovery), potential loss of income, and the desire to avoid further financial burden on his family. He, therefore, opted for conservative wound care, perceiving it as a lower-cost alternative.

After one month of conservative management failed to yield improvement, the ongoing costs of frequent clinic visits, coupled with the physical and psychological toll of the non-healing wound, led him to reconsider. Upon re-counseling regarding the potential malignant nature of the lesion and the risk of requiring more extensive and expensive treatment if progression occurred, he reassessed the risks and benefits. To achieve definitive disease control and prevent potentially higher long-term costs, he consented to the amputation.

Regarding the postoperative pathological evaluation, the patient explicitly declined additional immunohistochemical staining due to his severe financial limitations and the perception that curative resection had been achieved.

During follow-up, the patient expressed that while the loss of the toe was regrettable, he was satisfied with the resolution of chronic pain and infection. He reported an overall improvement in his quality of life post-operatively.

## 4 Discussion

PC is a rare malignant adnexal tumor of the skin. Its clinical presentation varies considerably and lacks specificity, often leading to diagnostic delay or misdiagnosis. To better understand the clinical significance of the present case, we systematically compared its key features with those of previously reported cases of pedal PC in the literature (**Table 1**). A synthesis of the literature indicates that pedal PC frequently manifests as a long-standing (several years) nodule, plaque, or ulcerative lesion. Definitive treatment typically involves surgical excision, including digital or limb amputation, with the disease demonstrating a potential for local recurrence and metastasis.

**Table 1:** Comparative analysis of clinical features between the present case of pedal eccrine porocarcinoma and cases reported in the literature.

PMID	(9)	(10)	(11)	(12)	(13)
Cases	1	1	1	1	2 (1 Ca, 1 Poroma)
Age/Sex	64, M	89, F	51, M	42, M	74, F
Location	Dorsum of foot	Dorsum of right foot and 4th toe	Plantar aspect of right heel	Plantar surface of right foot	Within scars on anterior/posterior right foot
Lesion Description (direct quotes)	“Raised, plaque-like lesions with a warty brown surface”; later “fleshy and erythematous”, lobular and ulcerated.	Three “brown, firm” nodules with crusting or ulceration, in stepping-stone distribution.	“A 3 cm, fungating, pedunculated lesion … focally eroded and irregularly nodular.”	Initially thought infectious. Later, “numerous growths over his entire body, some draining fluid.”	“Partly ulcerated, fresh, red nodule” (5 cm); “slightly elevated, reddish but partly white nodule” (4 cm).
Previous/Initial Diagnosis	Not specified.	Not specified.	Intraoperative frozen section: “infiltrating squamous cell carcinoma”.	The primary lesion was “initially thought to be infectious”.	Not specified.
Duration Before Diagnosis/Treatment	20 years	“Several years”	12 years	~2.5 years from diagnosis to death	Scars >50 years (post-TB spine injury)
Treatment	Excision and skin grafting	Surgical excision (x2); Topical 5-FU (x1)	Wide local excision (1 cm margin)	Excision; Multiple chemo/RT regimens for metastases	Not specified
Follow-up & Outcome	No recurrence or metastasis at 7 months.	No clinical evidence of metastasis at presentation. Follow-up outcome not reported.	No recurrence or metastasis at 2 years.	Died of widespread metastatic disease 2.5 years after initial diagnosis.	Not reported.
PMID	(14)	(15)	(16)	(17)	(18)
Cases	1	1	1	1	1
Age/Sex	39, F	71, F	67, F	72, F	73, M
Location	Plantar aspect, left foot (2nd met head)	Right heel	Left dorsum of foot	Lateral border of left foot	Subungual, right third toe
Lesion Description (direct quotes)	“A 1 cm firm, tender nodule … overlying integument was intact.” MRI: isointense nodule.	“A large verrucoid plaque containing a moist red nodule” (recurrence).	Primary not described in detail. In-transit metastasis: “reddish, beaded plaques on left lower leg.”	Porocarcinoma.	“Erythematous, partly eroded lesion associated with onycholysis … nail plate partially destroyed.” Misdiagnosed as onychomycosis/wart.
Previous/Initial Diagnosis	Radiologist suspected it was “either a wart or a benign fibrous lesion”.	Not specified.	Not specified.	Not specified.	Misdiagnosed and treated as “onychomycosis” and then as a “wart” for 10 years.
Duration Before Diagnosis/Treatment	2-3 months	1 year (recurrence after prior excision)	Primary excised 3 years prior; in-transit mets at 18 months	“Approximately five years”	10 years
Treatment	Re-excision with sentinel lymph node biopsy	Mohs micrographic surgery	Surgery + LN dissection + RT; then Cisplatin/5-FU for mets	Mohs surgery; reconstruction with keystone flap & V-Y plasty	Amputation of toe
Follow-up & Outcome	No recurrence at 18 months.	CT scan negative for metastasis 2 months post-op. Recurrence status not specified.	Complete remission of in-transit mets after chemotherapy (confirmed by PET-CT & biopsy).	No recurrence at 2 years.	Metastatic workup negative post-op. Long-term disease-free status not specified.
PMID	(19)			(20)	(21)
Cases	3			1	1
Age/Sex	79, F	73, F	34, F	87, F	62, M
Location	Heel	Forefoot plantar	Midsole	Dorsum of right foot	Fifth digit, left foot
Lesion Description (direct quotes)	“10-mm tan, slightly raised, friable lesion.”	“18-mm soft fleshy lobulated plaque.”	“8-mm reddish soft fleshy plaque.”	“Firm erythematous nodule.” Post-op wound: “8x6 cm, painful, malodorous, covered with adherent necrotic tissue.”	“A 1-cm, erythematous, fungating plaque.” Started as a blister.
Previous/Initial Diagnosis	Unknown prior to biopsy (diagnosed by punch biopsy 4 years earlier)	Provisional diagnosis: hemangioma (referred by dermatologist)	Not specified in text (diagnosis made by excision biopsy)	Not specified.	Initial biopsy diagnosed as “squamous cell carcinoma”.
Duration Before Diagnosis/Treatment	14 years	2 years	5 years	Excision in Aug 2015; wound complication 4 months post-brachytherapy (Oct 2015)	8 months (recurred after initial antibiotic)
Treatment	Complete excision	Complete excision	Complete excision	Excision + HDR brachytherapy; specialized wound care (UrgoClean Ag)	Amputation of digit
Follow-up & Outcome	No recurrence at 4-year follow-up.	No recurrence at 1-year follow-up.	No recurrence at 6-month follow-up.	Wound virtually healed at 12 months. Tumor-specific follow-up not reported.	No recurrence at 1-2 years follow-up.

In comparison to these established patterns, several aspects of our case are particularly notable. First, the patient was relatively young (51 years old), whereas pedal PC in the literature predominantly affects individuals over 60 years of age, with mean ages often reported above 67 years. Second, the lesion had a clear precipitating factor: it was directly initiated by minor local trauma (nail trimming) in the periungual region and progressed to a malignancy over a continuous, 9-month period of non-healing. This contrasts with the more common pattern described in the literature, where lesions are either “long-standing for many years” before malignant transformation or have an unclear origin. Although isolated case reports describe PC arising in old scars, reports of a malignancy being directly triggered by an acute minor trauma and progressing rapidly, as in this instance, are extremely rare. Third, the patient had a 30-year history of carbamazepine use. It is crucial to state that the association between carbamazepine and PC development in this case remains hypothetical and speculative, as no direct causal link has been established. Its potential immunomodulatory effects may have interacted with the post-traumatic chronic inflammatory milieu, creating a unique backdrop for tumorigenesis—an association not previously reported in the PC literature.

The notable feature of this case is the development of PC following minor trauma, with the wound failing to heal over an extended period. Existing literature has reported PC arising in areas of old scars or chronic trauma (22, 23). The underlying mechanisms may involve persistent inflammatory responses, accumulation of genetic mutations during repeated epithelial repair, and alterations in the local microenvironment. This case suggests that clinicians should maintain a high index of suspicion for any atypical, long-standing non-healing ulcers on the extremities. Even in the presence of a clear history of trauma, prompt biopsy is essential to rule out malignancy.

In this case, multiple factors may have collectively contributed to the chronification of the initial trauma. The acral location (toe) inherently exhibits relatively poor blood supply, which may have impaired healing. The patient’s underlying epilepsy must also be considered as a factor potentially contributing to a persistent wound environment. While no literature directly links epilepsy to tumorigenesis, the condition carries a known risk of accidental injury, including repetitive, unnoticed microtrauma to the extremities during seizures, which could have hindered the initial healing. Additionally, the wound received non−professional home care, which may have introduced contamination or inadequate debridement, thereby perpetuating inflammation. This situation created a classic “chronic wound” state.

Superimposed on this local microenvironment was the patient’s long−term use of carbamazepine. Although carbamazepine is not typically regarded as a direct cause of “delayed wound healing” in the conventional sense, its potent and sometimes pathological interactions with the immune system are well−documented. Most notably, carbamazepine is a known trigger of severe, life−threatening cutaneous adverse reactions such as Stevens–Johnson syndrome and toxic epidermal necrolysis (SJS/TEN), which are mediated by specific HLA alleles (24, 25). This demonstrates the drug’s capacity to provoke distinct immune−mediated pathological processes. Although none of the recognized severe immune−related adverse effects of carbamazepine [e.g., DRESS syndrome (26)] were observed, drug−induced subclinical alterations in immune status may have provided a predisposing condition for malignant transformation in the chronic wound. Laboratory findings at admission indirectly support this hypothesis. Testing indicated a potential state of immune dysregulation: decreased lymphocyte percentage (15.50%), vitamin D levels nearing deficiency (20.85 ng/mL), and hypoalbuminemia (34.44 g/L). Taken together, these markers suggest that drug−related subclinical immunosuppression superimposed on an underlying state of malnutrition may have weakened immune surveillance and normal wound−healing capacity, thereby promoting malignant transformation in the setting of chronic inflammation.

Thus, long−term medication leading to milder, subclinical immune modulation is a plausible speculation. Such potential immune changes could impair local immune surveillance and alter the wound microenvironment, an effect analogous to the “immunosuppression” listed among the risk factors for PC (27).

Mechanistically, carbamazepine is primarily metabolized by cytochrome P450 enzymes CYP3A4 and CYP3A5 (28). These enzymes are involved not only in drug metabolism but also in the biotransformation of endogenous compounds and may influence local tissue signaling. This provides a speculative mechanistic pathway through which chronic drug exposure might subtly modulate the inflammatory and immune landscape of a persistent wound. Their interaction with a chronic inflammatory state remains unexplored but is plausible. From a tumor biology perspective, PC itself is characterized by a squamous immunophenotype and frequent driver gene fusions (e.g., YAP1:MAML2/NUTM1), indicating that its pathogenesis is intrinsically linked to dysregulated cellular signaling and microenvironmental cues (5). Recent molecular studies on PC have highlighted alterations in pathways such as p53 and EGFR, and noted the presence of PD−L1, suggesting that immune escape may play a role in its progression (27).

Therefore, we propose a multifactorial hypothesis for this rare case. It is essential to acknowledge the patient’s epilepsy and overall health status as potential confounding factors that likely contributed to the chronicity of the wound. Drug−induced subclinical immune perturbations, converging with this local pro−inflammatory and proliferative chronic wound milieu—itself sustained by acral anatomy, potential microtrauma, and suboptimal care—may have collectively created a permissive environment for malignant transformation. This hypothesis integrates clinical observations with existing evidence and emphasizes the need for further research into the oncogenic potential of chronic inflammation, particularly in patients on long-term systemic medications that may have immunomodulatory properties.

Diagnosing PC remains challenging due to its nonspecific clinical and histologic features. It typically presents as an erythematous nodule or ulcer, resembling conditions like chronic ulcers, pyogenic granuloma, or squamous cell carcinoma (SCC), often resulting in delayed diagnosis (29). Histologically, while well-differentiated PC shows characteristic patterns, small biopsies or poorly differentiated variants can closely mimic metastatic adenocarcinoma, SCC, or other adnexal tumors, creating diagnostic difficulty (6). Ancillary tools are therefore essential. Conventional immunohistochemical markers like EMA and CEA lack specificity for reliably distinguishing PC from SCC (6). Recent advances in molecular pathology have identified YAP1-NUTM1 or YAP1-MAML2 fusions as highly specific for poroid neoplasms, aiding differentiation from SCC or Merkel cell carcinoma (MCC) (30, 31). Additionally, retained RB1 protein expression helps differentiate PC from SCC and MCC, which frequently show RB1 loss (30).

Managing PC requires balancing oncologic control with functional preservation. Wide local excision (WLE) is standard for localized disease, yet local recurrence rates after WLE approach 20% (6). Mohs micrographic surgery (MMS) allows more precise margin assessment and may lower recurrence rates, though its use can be limited by technical and anatomical constraints (32). In this case, the deep-seated toe location led to choosing amputation as a definitive option to secure clear margins and minimize recurrence risk. This approach also accounted for the patient’s epilepsy, avoiding prolonged flap reconstruction and associated perioperative risks. Lymph node metastasis rates for PC range from 3.7% to 21.4%, reflecting its aggressive potential (33). No consensus exists on sentinel lymph node biopsy (SLNB), though it is often considered for high-risk features such as tumor depth ≥3 mm (6, 34, 35). Based on initial assessment, SLNB was not performed here, aligning with prevailing practice. For advanced metastatic PC, evidence remains limited, though immune checkpoint inhibitors and EGFR inhibitors represent potential therapeutic pathways (6).

## 5 Conclusion

In summary, PC poses a significant diagnostic challenge due to its rarity and its propensity to mimic a wide spectrum of benign or common malignant skin conditions, often leading to delayed diagnosis. This case underscores the importance of maintaining a high index of suspicion for PC when evaluating refractory, atypical ulcers in acral locations, irrespective of a documented traumatic incident. A low threshold for early biopsy is recommended for definitive diagnosis. Additionally, the long-term carbamazepine use in this patient suggests that systemic medication-induced, subclinical immunomodulation may synergize with the chronic inflammatory microenvironment created by local trauma, potentially contributing to oncogenesis. However, the precise mechanisms and causal relationships between drug-induced immune alterations, trauma, and the development of PC remain elusive and warrant further investigation. Finally, while our patient showed no signs of recurrence at the 6-month follow-up, this period remains relatively short for a malignancy with known potential for late recurrence and metastasis. Therefore, prolonged clinical surveillance beyond 12 months is strongly advised for similar cases to better understand the long-term outcome.

## 6 Study limitations

This case report has certain limitations that should be acknowledged. First, the postoperative follow-up period of 6 months, while indicating a favorable short-term outcome, is insufficient to fully assess the long-term oncological prognosis of PC, which can recur or metastasize years after initial treatment. Extended follow-up is needed to draw definitive conclusions regarding cure. Second, the definitive histopathological diagnosis relied on morphological examination (H&E staining) without supplementary immunohistochemical analysis. This was due to the patient’s explicit decision, based on severe financial constraints, to forgo additional costly testing after curative resection was deemed achieved. While the morphological features were highly suggestive of eccrine porocarcinoma and all surgical margins were clear, immunohistochemistry could have provided further confirmatory evidence and potentially yielded additional biomarker information relevant to the tumor’s biology.

## Data Availability

The raw data supporting the conclusions of this article will be made available by the authors, without undue reservation.
